# Lysosome activable polymeric vorinostat encapsulating PD-L1KD for a combination of HDACi and immunotherapy

**DOI:** 10.1080/10717544.2021.1927246

**Published:** 2021-05-26

**Authors:** Fengkun Lu, Lei Hou, Sizhen Wang, Yingjie Yu, Yunchang Zhang, Linhong Sun, Chen Wang, Zhiqiang Ma, Feng Yang

**Affiliations:** a Department of Pharmacy, Hebei North University Hebei Key Laboratory of Neuropharmacology, Zhangjiakou, People’s Republic of China; b School of Pharmacy, Second Military Medical University, Shanghai, People’s Republic of China; c Department of Oncology, Ruijin North Hospital, Shanghai Jiao Tong University School of Medicine, Shanghai, People’s Republic of China

**Keywords:** Histone deacetylase inhibitor (HDACi), HDACi prodrug, immunotherapy, siRNA-PD-L1, PD-L1KD

## Abstract

PD-1/PD-L1 blocking therapy has become one of the most promising methods in the field of tumor treatment. However, it encounters the challenge of immune escape due to the exhaustion of T cells. Studies have shown that the epigenetic regulation drug histone deacetylase inhibitor (HDACi) may be able to reverse exhausted T cells by changing the epigenetic transcription program. Therefore, the combination of epigenetic therapy and PD-1/PD-L1 blockade therapy is expected to reverse the immune escape, whereas the overriding goal should aim at the spontaneous release and synergy of PD-1/PD-L1 blocking siRNA and HDACi. In this study, we develop PDDS{polyethylene glycol-b-asparaginate(diethylenetriamine-vorinostat), (PEG-b-P[Asp(DET-SAHA)_n_] PPDS)}encapsulating siRNA-PD-L1to provide micelles siRNA-PD-L1-loaded micelles (siRNA@PPDS). Transmission electron microscope (TEM) images demonstrate that siRNA@PPDS micelles presented spherical morphology with a size of about 120 nm; hydrodynamic data analysis indicates pH sensitivity of siRNA@PPDS micelles. The experiments reveal that siRNA@PPDS micelles could be well uptaken by the tumor cells to silence the expression of PD-L1 protein in a dose-dependent manner; compared with the free SAHA, the SAHA-loaded micelles PPDS show higher cytotoxicity to induce tumor cell apoptosis and block cell cycle in G1 phase on melanoma-bearing mice, siRNA@PPDS has shown outstanding inhibition of tumor growth and pulmonary metastasis. By comprehensively activating the immune system, lysosome activable polymeric vorinostat encapsulating PD-L1KD for the combination therapy of PD-L1-KD and HDACIs can be an effective strategy to reverse the unresponsiveness of immune checkpoint inhibitors and a promising treatment to inhibit tumor growth, recurrence, and metastasis in clinic.

## 1. Introduction

Tumor cells avoid the immune response in a process known as immune evasion (Schreiber et al., 2011; Zhou et al., 2020; Ren et al., 2021; Zhou et al., 2021). The immune escape of tumor cells is divided into three stages: (1) Elimination phase: at this stage, antigen-presenting cells (APC) monitor the antigens expressed by tumors and present them to cytotoxic T cells through major histocompatibility complex (MHC) class I molecules, which is the first signal of T cell activation, then the molecule CD80/CD86 on the surface of APC combines the costimulatory molecule CD28 on the surface of T cells which forms a costimulatory signal (Murakami & Riella, 2014), thereby cytotoxic T cells are activated, they target and eliminate tumor cells by secreting perforin, granzyme or by the death ligand/death receptor pathway (Martinez-Lostao et al., 2015). (2) Balance and control phase: at this stage, tumor cells gradually become resistant to immune surveillance. Due to the influence of the tumor microenvironment, tumor cells and some immunosuppressive cells gradually exhibit mechanisms of immune suppression. For example, they will overexpress programmed death ligand-1 (PD-L1), a ligand for the programmed death receptor-1 (PD-1) protein on cytotoxic T cells, the combination of PD-1 and PD-L1 forms a co-inhibitory signal for T cell activation and inhibits immune response. It may last for several years. (3) Escape stage in the tumor microenvironment, tumor cells and some immunosuppressive cells such as regulatory T cells will express more immunosuppressive receptors such as PD-L1, which bind PD-1 to the surface of T cells to form a cosuppressive signal. As a result, T cells cannot be activated, showing a state of exhaustion and losing their ability to kill tumor cells. Tumor cells thus evade the immune response and proliferate uncontrollably, leading to clinically detectable tumors.

In order to solve the problem of immune escape, immunotherapy against immune checkpoints has attracted widespread attention. In recent years, the therapy of immune checkpoint inhibitors (ICIs) has become one of the most promising strategies in the field of cancer treatment. Compared with chemotherapy and radiotherapy targeting tumor cells, ICIs directly restore the exhausted host antitumor immune responses mediated by the tumor microenvironment. Under chronic stimulation of tumor antigens, CD8+ T cells are progressively exhausted, which is related to a unique transcription program that up-regulates the expression of T cell surface inhibitory receptors (IRs) such as PD-1 and cytotoxic T lymphocyte-associated protein-4 (CTLA-4). In patients suffering from Hodgkin’s lymphoma, melanoma, hepatocellular carcinoma, and gastric cancer, the expression of PD-1 on tumor-infiltrating CD8+ T cells was significantly up-regulated (Yamamoto et al., 2008; Saito et al., 2013). Moreover, most tumor cells overexpress PD-L1 in the tumor microenvironment. In this situation, ICIS such as PD-1/PD-L1 inhibitors stand out to block the co-inhibitory signal by hindering the binding of PD-1 and PD-L1torejuvenate T cell (Xia et al., 2019). This discovery was a breakthrough in the field of tumor immunotherapy (Xia et al., 2016), making PD-1/PD-L1 immune checkpoint the most promising target for cancer drug discovery and development (Liu et al., 2017). Therapeutic monoclonal antibodies against PD-1 or PD-L1 have shown significant clinical efficacy in the treatment of various advanced tumors (Kline & Gajewski, 2010; Liu et al., 2017). Up to now, the US Food and Drug Administration (FDA) has approved 5 monoclonal antibodies against PD-1 or PD-L1 for the treatment of various advanced tumors, including melanoma (Bhatnagar et al., 2017), non-small cell lung cancer (Kazandjian et al., 2016), squamous cell carcinoma of the head and neck (Larkins et al., 2017), classic Hodgkin lymphoma (Kasamon et al., 2017), urothelial cancer (Ning et al., 2017), hepatocellular carcinoma (Bhatnagar et al., 2017), merkel cell carcinoma (Kim, 2017), renal cell carcinoma (Xu et al., 2017), and colorectal cancer (Sclafani, 2017).

Despite unprecedented clinical success, a large proportion of cancer patients did not show an objective response after ICIS treatment (Sharma & Allison, 2015), some patients who initially respond to ICIS treatment relapsed (Ribas et al., 2016). The mechanism of unresponsiveness and recurrence caused by ICIS treatment is: (1) ICIS cannot activate severely exhausted T cells that exist in the immunosuppressive tumor microenvironment (Youngblood et al., 2011; Zhang et al., 2014; Pauken et al., 2016); (2) Partially exhausted T cells can be reactivated, but due to stable changes in their epigenetic program (Youngblood et al., 2011; Zhang et al., 2014; Ghoneim et al., 2016; Pauken et al., 2016), they cannot maintain their ability to express cytokines and kill tumor cells for a long time, leading to recurrence. Studies have found that epigenetic therapy can reverse the resistance of ICIS treatment in many ways (Schmidl et al., 2018). Each step of the immune process (1) Antigen presentation and T cell activation; (2) T cells transport and infiltrate into the tumor; (3) T cells recognize and remove tumor cells) can be regulated by epigenetic therapy, destroying the immunosuppressive state (He et al., 2016; Im et al., 2016). Epigenetic therapy combined with ICIs can restore immune recognition and eliminate tumors to increase the clinical response rate (Heninger et al., 2015). Epigenetic regulation drugs, which include DNA methyltransferase inhibitor (DNMTi), histone deacetylase inhibitor (HDACi) and histone methyltransferase inhibitor (HMTi), can stimulate antitumor immunity of both tumor cells and host immune cells at the same time. HDACi shows selective cytotoxicity to tumor cells, for it can mediate different biological responses that affect tumor cell development, growth, survival and immunogenicity, therefore, HDACi can enhance the body's response to anti-PD-1 treatment. Specifically, on the one hand, HDACi can inhibit tumor cell growth and survival. HDACi increases the expression level of tumor suppressor genes such as p21by increasing the degree of acetylation of histones in the cell (Dokmanovic et al., 2007; Di Pompo et al., 2015; Fu et al., 2019; Huang et al., 2019), thereby inducing tumor cell cycle arrest, differentiation, and apoptosis. On the other hand, HDACi inhibits tumor immune escape. Epigenetic reprogramming is a key mechanism to promote tumor cells escape, because the expression of cell surface molecules necessary for the immune system to recognize and eliminate tumor cells were silenced or down-regulated epigenetically, such as specific tumor-associated antigens (TAA), human leukocyte antigens (HLA) and co-stimulatory molecules (Campoli & Ferrone, 2008; Fratta et al., 2011; Vesely et al., 2011; Escors, 2014; Sigalotti et al., 2014), and epigenetic drugs can inhibit tumor cell immune escape by up-regulating the expression of these genes (Maeda et al., 2000; Mizukami et al., 2008). Therefore, the combination of epigenetic agent HDACi and immunotherapy can be considered as a potential idea in cancer treatment. All the above research provides a basis for the combined use of HDACi and PD-L1 knock down (PD-L1 KD) therapy.

In this study, we hypothesized that siRNA blocking PD-1/PD-L1 pathway-induced cancer immunotherapy could be augmented by SAHA, a pan HDACIs. Nevertheless, HDAC is are usually of low solubility, cellular permeability, and short half-life *in vivo* (*t*_1/2_ of SAHA was only 0.8–3.9 h), which leads to an inadequate therapeutic effect; more seriously, siRNA is easily degraded without proper protection during the circulation due to RNase. Therefore, a direct combination of those two drugs leads to an inadequate synergistic effect and complicated pharmacokinetic issues, hence in, it is an urgent demand to fabricate a drug co-delivery system to realize a highly efficient combination of HDACi and siRNA-PD-L1 to realize a synergistic antitumor effect.

**Scheme 1.**
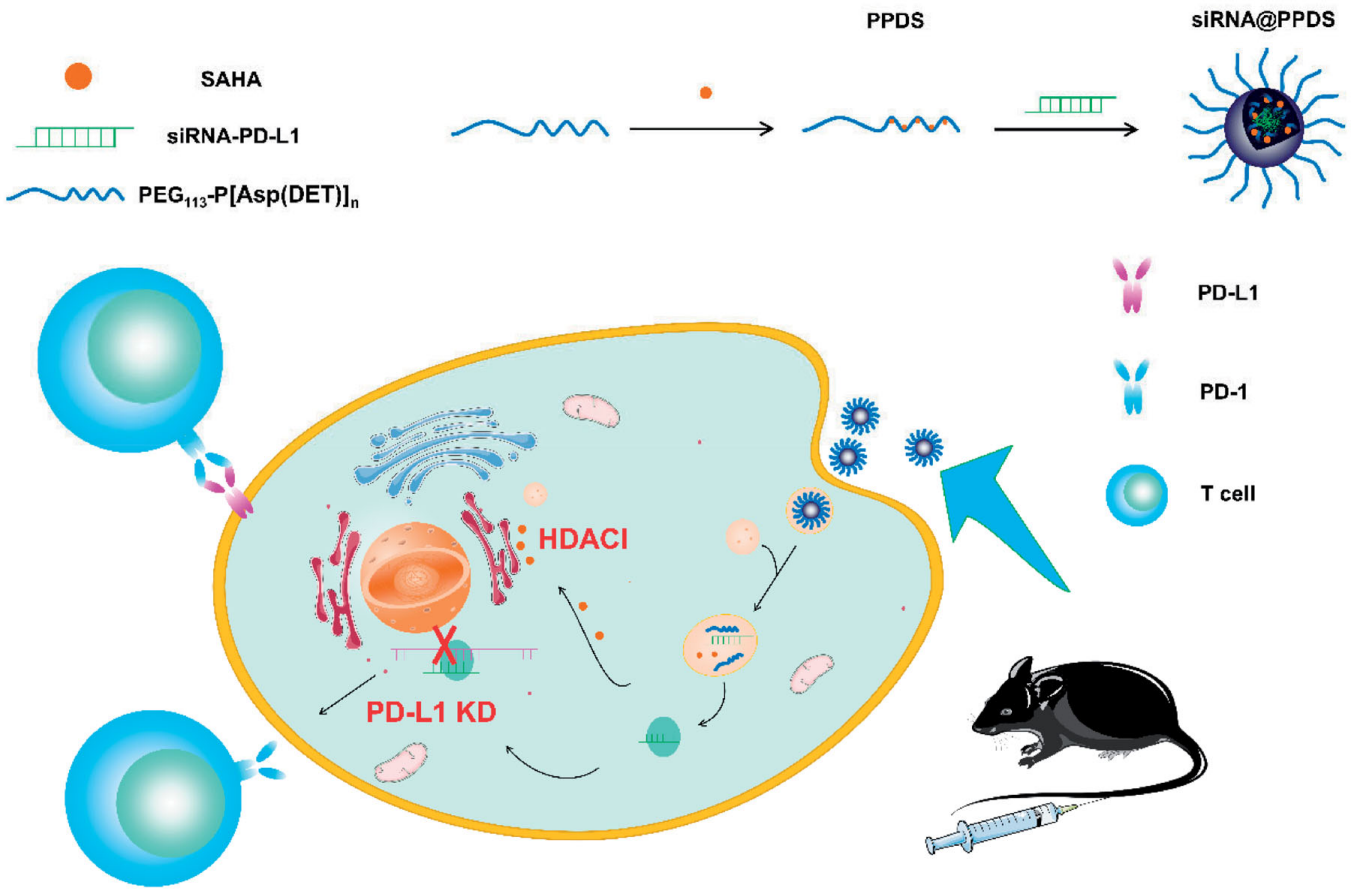
Illustration of self-assembly and lysosome activable of siRNA@PPDS. The self-assembly lysosome activable micelles siRNA@PPDS disintegrated at pH5 in the lysosome after being cellularly uptaken, then, siRNA and SAHA were simultaneously released to exert the synergistic anti-tumor effect of PD-KD and HDACI.

To verify our hypothesis, we synthesized an acid-activatable micelles co-loaded with SAHA and siRNA-PD-L1 for inhibiting immune escape and enhancing cancer immunotherapy. As is superior to antibody, siRNA can down-regulate the expression of both the membrane and cytosolic protein by degrading endogenous mRNA, while the former neutralizes membrane protein only (Dolina et al., 2013). The micelles were composed of three independent components. As shown in Scheme 1, the matrix of micelles was a pH-responsive block copolymer polyethylene glycol-b-poly-asparagine [PEG-b-P[Asp(DET)*_n_*] (PPD)] with remarkably low toxicity, facilitating its use for drug delivery (Kanayama et al., 2006). PPD can achieve co-loading and intracellular release through lysosomes because of the unique properties of the ethylenediamine unit (DET) integrated into the poly aspartamide side chains. Apan HDACI vorinostat (SAHA) was grafted onto the DET through an ester bond to form polyethylene glycol-b-polyasparagine-vorinostat [PEG-b-P[Asp(DET-SAHA)*_n_*] (PPDS)], a HDACi prodrug releasing responsive to the lysosome fluid with the lipase; As the DET segments in PPDS presented a mono-protonated form at pH 7.4, PPDS could encapsulate negatively charged siRNA-PD-L1 to self-assembly the final stable complex siRNA-PD-L1-loaded micelles (siRNA@PPDS). The lysosome activable micelle complex maintained inert at physiological pH conditions and could be activated with internalization into lysosome of the tumor cells. Specifically, after siRNA@PPDS being taken up by the cell, since the micelles could be disintegrated at ∼ pH 5.3 in the lysosome, the siRNA was released into the cytoplasm to reverse the exhaustion of T cells by silencing the PD-L1 protein of tumor cells to activate the killing function of the immune system against tumors; simultaneously, SAHA was also released due to the lipase catalyzing ester bond breaking between the PPD and SAHA to stimulate antitumor immunity of both tumor cells and host immune cells. Theoretically, the micelles could reverse PD-L1 KD resistance by activating the immune system more comprehensively, inducing tumor cell cycle arrest and apoptosis, which were verified in the following experiments.

## 2. Materials

L-aspartate-4-benzyl ester (H-Asp(OBzl)-OH), amino polyethylene glycol (PEG-NH_2_, MW = 5000), Tetrahydrofuran (THF), formamide (DMF), N,N’-carbonyldiimidazole(CDI), triphosgene, Vorinostat (SAHA) and N,N-dimethylformamide (DET) were purchased from Shanghai Aladdin Bio-Chem Technology Co., Ltd. (Shanghai, People’s Republic of China). N,N-dimethyl ethylacetate, dichloromethane (DCM), and dimethyl sulfoxide (DMSO) were purchased from Chinese Medicine Group Chemical Reagent Co., Ltd. (Shanghai, People’s Republic of China). siRNA targeting PD-L1 labeled with cy3 (sense: 5′-GGAUAUUUGCUGGCAUUAUTT-3′, antisense: 5′-AUAAUGCCAGCAAAUAUCCTT-3′; sense: 5′-GAAGGGAAAUGCUGCCCUUTT-3′, antisense: 5′-AAGGGCAGCAUUUCCCUUCTT-3′; sense: 5′-GGAGCUGAUCAUCCCAGAATT-3′, antisense: 5′-UUCUGGGAUGAUCAGCUCCTT-3′) and siRNA-NC or siRNA-NC labeled with cy3 were purchased from Gene Pharma of Shanghai.

## 3. Methods

### 3.1. Preparation of PPDS

Polyethylene glycol-b-polyasparagine [PEG-b-P[Asp(DET)*_n_*] (PPD)] was synthesized according to the reference literature (Kanayama et al., 2006). Briefly, 100 mg SAHA was dissolved in DMF, and 62 mg CDI in DMF was mixed and stirred magnetically for 1 h under the protection of N_2_ atmosphere at room temperature, then 186 mg PEG_113_-P[Asp(DET)*_n_*] was added to the mixture solution and magnetically stirred for 24 h in the N_2_ atmosphere at 35 °C. The crude product was dialyzed against deionized water at pH 5 for 24 h and lyophilized for 3 days to obtain the final product PPDS as yellow powder. The conjugation of PPD with SAHA was demonstrated by ^1^HNMR spectrum and SAHA grafting ratio (n_SAHA_:n_terminated amino_)was determined by the ratio of the peak areas of the corresponding groups. The specific calculation method was described in the Supplementary materials.

### 3.2. Optimizing the N/P of siRNA@PPDS

The agarose gel electrophoresis assay was used to evaluate the loading capability of PPDS with siRNA. To optimize the N/P ratio (PPDS to siRNA-PD-L1) for highly transfection of siRNA, the stability of siRNA@PPDS *in vitro* were measured by agarose gel electrophoresis. Briefly, siRNA@PPDS micelles were prepared at different N/P ratios (0, 8, 16, 24, 32, 40), then they were loaded in an agarose gel (1%), followed by electrophoresis at 100 V for 30 min.

### 3.3. Characterization of siRNA@PPDS

The siRNA@PPDS micelles were dyed by uranyl acetate and observed under transmission electron microscope (TEM) (TecnaiG2 F20 S-Twin; FEI, Hillsboro, OR). The hydrodynamic size and diameter distribution of siRNA@PPDS micelles at pH 5 and pH 7.4 were measured by dynamic light scattering (DLS, ZEN3600, Malvern Panalytical, UK).

### 3.4. Lipase catalyzed SAHA release

The release profiles of SAHA from PPDS were conducted using a simulated lysosome juice. Briefly, 13.5 mg PPDS was dissolved in 2 mL release medium (PBS:DMSO = 99:1). 0.5 mg of lipase was added to 1 ml of PPDS solution and the mixture was transferred into a dialysis tube. The rest 1 ml PPDS solution was added to the other dialysis tube without lipase, then the dialysis bags were immersed in 20 mL of release medium, respectively, and were put in a constant temperature shaker (37 °C, 100 rpm). The absorbance of the released SAHA was measured by UV/VIS at 0.5, 1, 2, 3.5, 5.5, 10, 27, 35, 48, 59 h, the cumulative release amount was calculated by SAHA standard curve (Supplementary Figure S2) to draw the cumulative release curve.

### 3.5. Cellular uptake assay

B16-F10 cells with a concentration of 10^5^/mL were seeded on confocal plates for 12 h. The growth medium was then replaced by mediums with 200 nM free siRNA-PD-L1 or siRNA@PPD. After incubating for 12 h, all the cells in each plate were washed by PBS for three times and fixed by 4% paraformaldehyde for 20 min, then, the nucleus was stained with 2-(4-Amidinophenyl)-6-indole carbamidine dihydrochloride (DAPI) for 3 min. At last, the cells were washed by PBS for three times, dispersed in 0.5 mL of PBS and observed under a confocal laser scanning microscope (CLSM, ZEISS710, Germany).

### 3.6. Transfection efficiency of siRNA-PD-L1 in vitro

Western blotting experiments were used to detect the transfection efficiency by measuring PD-L1 expression level of siRNA@PPD on B16-F10 cells. Briefly, B16-F10 cells (2 × 10^5^ cells/well) were seeded on six-well plates and incubated overnight at 37 °C, then the culture medium was removed and replenished with siRNA@PPD (25 nM, 50 nM,100 nM, 200 nM of siRNA-PD-L1) and siRNA-NC@PPD (200 nM of siRNA-negative controls) for 24 h. All the cells were collected and lysed to harvest the proteins, and then the concentrations of proteins were measured by the western blotting experiments.

### 3.7. Cell viability assay

The cytotoxicity of PPDS against B16-F10 and HepG2 was detected *via* Cell Counting Kit-8 (CCK-8). Briefly, the cells (10^4^ cells/well) were cultured in 96-well plates and incubated for 12 h. For B16-F10 cells, the growth medium was then replaced with formulations containing free SAHA or PPDS (1.25, 2.50, 5.00, 10.00, 20.00, 40.00, 80.00, 160.00, and 320.00 μM of SAHA) for 48 h. For HepG2 cells, the growth medium was then replaced with formulations containing free SAHA or PPDS (0.15625, 0.3125, 0.625, 1.25, 2.5, 5, 10 μM of SAHA) for 48 h. Cells were then washed and incubated with100 μL of fresh culture medium with 10%CCK-8 for 2 h at 37 °C. The absorbance of each well was observed by a microplate reader (EL × 800; BioTek, Winooski, VT) at 450 nm to assess the cell viability by CCK-8 assay (Dojindo Laboratories, Japan) according to the manufacturer’s protocol.

### 3.8. Apoptosis assay

As B16-F10 cells could absorb fluorescence due to their own pigments which severely effect the determination of fluorescence, herein, HepG2 cells were chosen to evaluate PPDS induced apoptosis with FITC-AnnexinV/PI kit. Briefly, HepG2 cells (2 × 10^5^cells/well) were seeded into six-well plates and incubated overnight at 37 °C, then the culture medium was removed and replenished equal volume of culture medium with free SAHA (1.25 μM and 2.5 μM) or PPDS (at an equivalent concentration to SAHA). After incubating for 48 h, all the cells were collected and rinsed twice with PBS to investigate the apoptosis of HepG2 cells with FITC-AnnexinV/PI by flow cytometry (BD FACS Calibur, USA).

### 3.9. Cell cycle assay

EZ Cell TM Cell Cycle Analysis Kit was used to evaluate cell cycle arrest of B16-F10 cells exposing to PPDS. Briefly, B16-F10 cells (2 × 10^5^ cells/well) were seeded into six-well plates and incubated overnight at 37 °C, then the culture medium was removed and treated with free SAHA (20 and 40 μM) or PPDS (at a concentration equivalent to SAHA). After incubating for 24 h, all the cells were collected and rinsed twice with PBS. Finally, the cells were collected to investigate cell cycle arrest with Cell Cycle Analysis Kit by flow cytometry.

### 3.10. Antitumor activity in vivo

1 × 10^5^ B16-F10 cells were subcutaneously injected into each C57BL/6 mouse to prepare melanoma-bearing mouse models to evaluate the antitumor efficacy of siRNA@PPDS micelles. When the tumor volume reached about 40 mm^3^, the mice were randomly divided into four groups (*n* = 6): saline group (negative control), siRNA@PPD group, PPDS group and siRNA@PPDS group. The mice were iv-injected with 200μL of saline, siRNA@PPD, PPDS or siRNA@PPDS at a SAHA and siRNA-PD-L1 dose of 7 and 1.98 mg·kg^−1^, respectively. Injection were conducted in five cycles at a time interval of 2 days. Tumor volume and the survival rate were monitored during the procedure. The tumor volume was calculated by formulation V = L × W × W/2 (L, the longest dimension; W, the shortest dimension). To investigate the antimetastasis effect of siRNA@PPDS, we observed the metastasis of lung tissue nodules as follows: the intact lung from the first dead mice in every group was harvested, photographed, and fixed in 4% paraformaldehyde, then the lung tissue was paraffin embedded, sectioned, and stained with hematoxylin and eosin (H&E) for microscopic analysis (Olympus CKX41A22PHP).

### 3.11. Statistical analysis

The statistical significance of tumor volume between any two groups was conducted in GraphPad Prism 5.0 by one-way ANOVA. The mice survival curve was analyzed by Log-rank (Mentel of Cox) Test. A *p*-value below .05 was considered to be statistically significant for all analyses.

## 4. Results and discussion

### 4.1. Preparation and characterization of siRNA@PPDS

**Figure 1.**
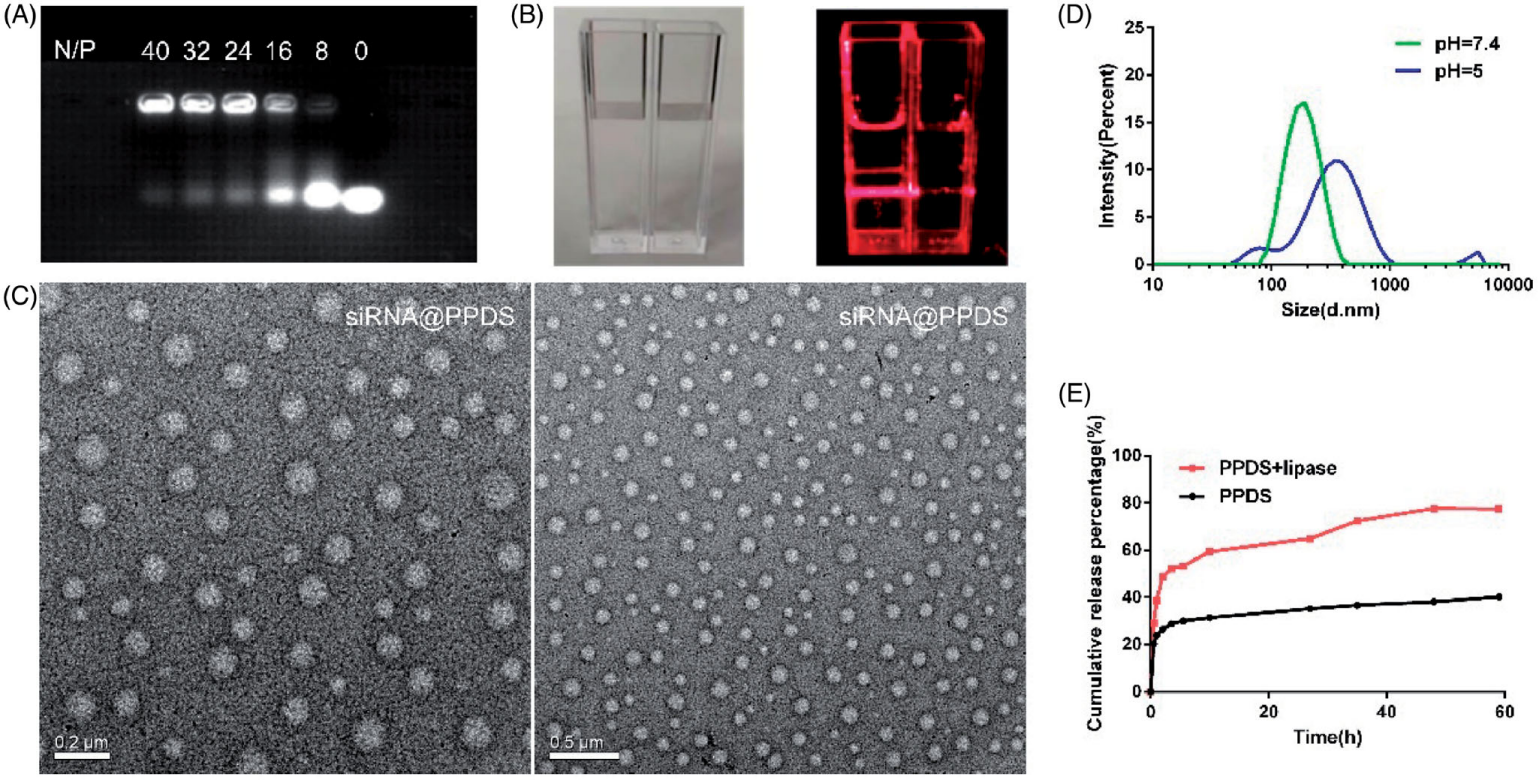
Preparation, characterization, release performance of PPDS. (A) Agarose gel retardation of siRNA@PPDS at various N/P ratios. (B) The Tyndall phenomenon of siRNA@PPDS. Left inset: siRNA@PPDS and water without laser irradiation; right inset: siRNA@PPDS and water with laser irradiation. (C) TEM image of siRNA@PPDS. Scale bar = 0.2 μm, 0.5 μm, respectively. (D) Hydrodynamic diameter distribution of siRNA@PPDS by DLS at pH = 7.4 or pH = 5. (E) Lipase-responsive release curve of SAHA in simulated lysosome juice in 60 h.

The synthetic route (Supplementary Figure S1-1), ^1^HNMR spectrum of PPD (Supplementary Figure S1-2A) and PPDS (Figure S1-2B) were shown in Supplementary materials. The appearance of benzene ring peak of SAHA at *δ* = 7.0–7.5 ppm in PDDS spectrum indicated the conjugation of PPD with SAHA; The migrations of PPDS bound siRNA in gel electrophoresis were thoroughly blocked at the ratio of 40:1 (Figure 1(A)), indicating PPDS strongly bound siRNA could resist dissociation during drug delivery *in vivo*, The N/P ratio 40 was adapted in the following investigation. Correspondingly, Tyndall phenomenon was observed in a dark atmosphere at N/P ratio 40, showing the siRNA@PPDS micelles well dispersed in the solution (Figure 1(B)); TEM images demonstrated siRNA@PPDS micelles spherical morphology with a size of about 120 nm (Figure 1(C)). The particle size inspected by dynamic light scattering (DLS) showed a size of 181.9 nm and a narrow size distribution (PDI, of ∼0.244) at pH 7.4, while the particle size increased to ∼272.6 nm at pH 5 with a wide size distribution (PDI, of ∼0.363), indicating a looser structure of siRNA@PPDS micelles (Figure 1(D)), the pH sensitivity of the micelles played a crucial role in the pH responding release of siRNA in cytoplasm.

### 4.2. Determination of lipase triggered SAHA release in vitro

By simulating normal physiological environment and the endosomal/lysosomal microenvironments in tumor cells, SAHA release from PPDS was investigated in two buffer solutions (with lipase or without lipase) at 37 °C using the dialysis method. As shown in Figure 1(E), in the presence of lipase, 77.68% of SAHA was released from PPDS at 48 h, in contrast, with the absence of lipase, only 38.14% of SAHA was released, indicating that accumulative release of SAHA was lipase-dependent, as at the physiological conditions of the lysosome, the structure of micelles probably become looser or even disassembly, the ester bond linking SAHA and PPD exposed to the lipase in the cytoplasm, leading a simultaneously release of siRNA and SAHA in the cytoplasm.

### 4.3. Cellular uptake of siRNA@PPD

**Figure 2.**
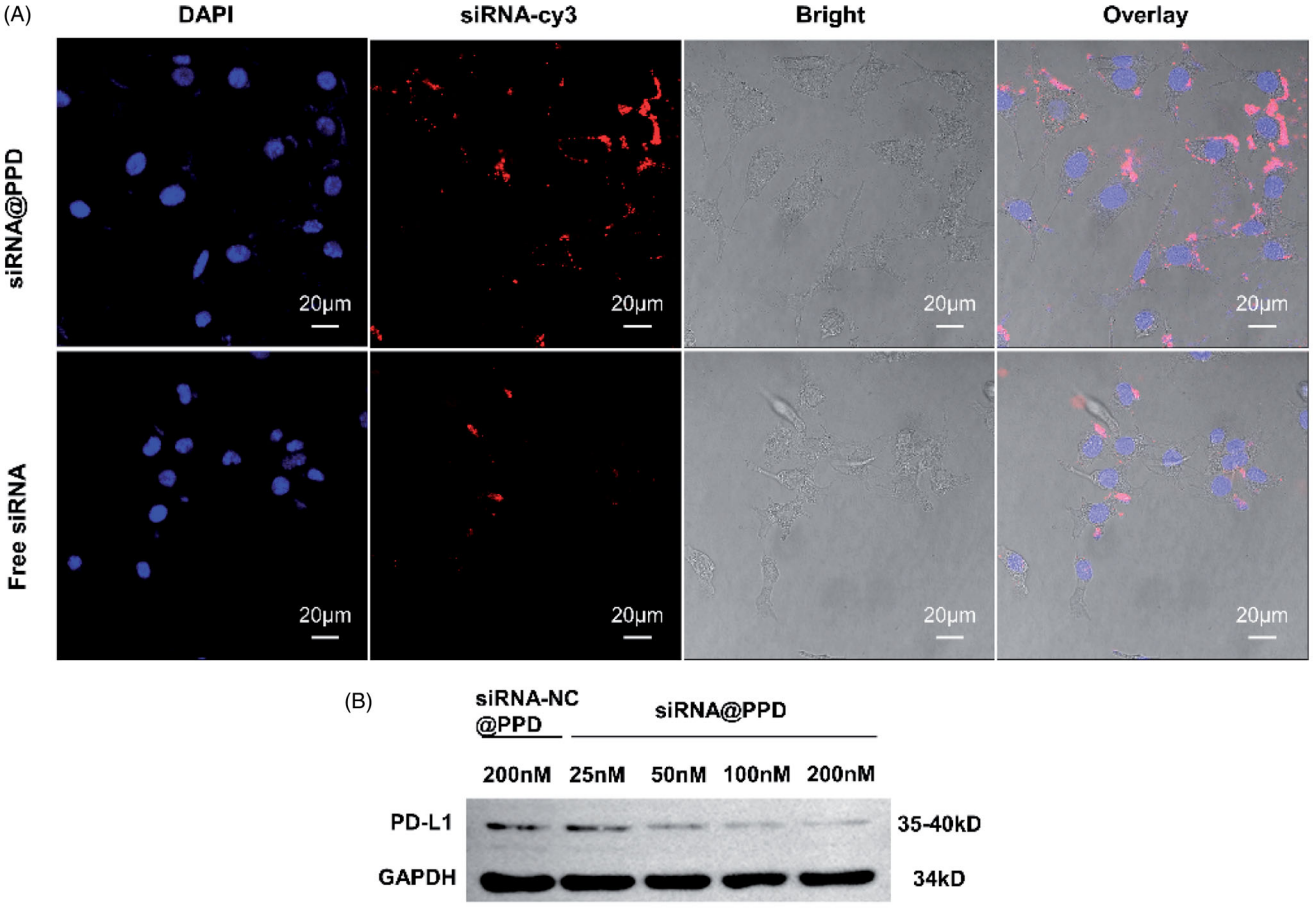
(A) Cellular uptake of siRNA@PPD. Fluorescence images of B16-F10 cells under laser confocal microscopy after co-incubation with 200 nM of siRNA@PPD and free siRNA for 12 h. Scale bar = 20 μm. The cell nucleus was stained by DAPI emitted blue fluorescence, and siRNA-cy3 uptake by cells emitted red fluorescence. (B)PD-L1protein silencing of siRNA-PD-L1 *via* western blot *in vitro*. PD-L1 protein silencing effects of siRNA@PPD (25 nM, 50 nM, 100 nM, 200 nM of siRNA-PD-L1) inB16-F10 cells detected by Western blot analysis.

The intracellular uptake and distribution of siRNA@PPD micelles in B16-F10 cells were examined using CLSM. Confocal images (Figure 2(A)) displayed that siRNA@PPD micelles delivered siRNA-cy3 with red fluorescent labeling were visible throughout the cytosol, and the intensity of red fluorescent labeling was obviously greater than that of free siRNA. It indicated that siRNA@PPD micelles were more efficiently taken up by B16-F10 cells and mainly stayed in the cytoplasm after 12 h of incubation, confirming the efficient cellular uptake of siRNA@PPD micelles.

### 4.4. Downregulation of PD-L1 expression

Western blotting assay was used to determine the PD-L1 protein level in B16-F10 cells. siRNA@PPD micelles were prepared for PD-L1 KD protein silencing. While PPD loaded with siRNA-NC (200 nM) were utilized as a blank control. As shown in Figure 2(B), siRNA@PPD micelles significantly suppressed PD-L1 expression in B16-F10 cells in a dose-dependent manner. In particular, when the concentration of siRNA-PD-L1 was 100 nM and 200 nM, the expression of PD-L1 protein was down-regulated to about 66 and 24%, respectively, demonstrating siRNA@PPD micelle could guarantee siRNA-PD-L1 entering the B16-F10 cells and performing its function of silencing PD-L1 protein. In addition, the siRNA-NC or siRNA-PD-L1 loaded micelles (siRNA-NC@PPD or siRNA@PPD) showed comparable biocompatibility in B16-F10 cells (Supplementary Figure S3A), suggesting that PD-L1 KD negligibly affected the proliferation of tumor cells without T-cell-mediated.

### 4.5. *In vitro* cytotoxicity study

**Figure 3.**
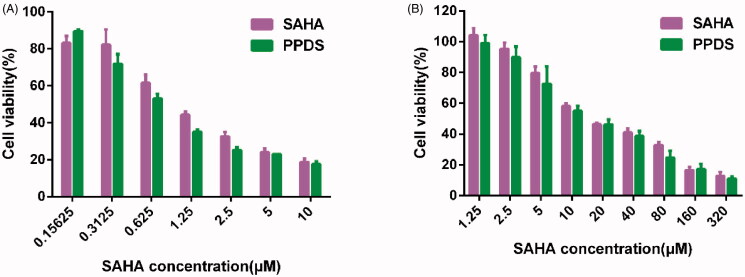
Cytotoxicity of PPDS *in vitro*. Relative viabilities of HepG2 (A) and B16-F10 (B) cells were analyzed by CCK-8 assay after treatment by various concentrations of PPDS and free SAHA.

CCK-8 assay was employed to investigate anticancer activity of PPDS micelles *in vitro*. The results showed that the PPD micelles were less toxic toHepG2 cells (Supplementary Figure S3B) or B16 F10 cells (Supplementary Figure S3C), while PPDS and free SAHA severely inhibited the growth of tumor cells in a dose-dependent manner after being cultured for 48 h. The half maximal inhibitory concentration (IC50) values were calculated according to above cell relative viabilities (Supplementary Table 1). For HepG2 cells (Figure 3(A)), the IC50 value of PPDS was 0.8091 μM, which was lower than IC50 value of free SAHA (1.129 μM). For B16 F10 cells (Figure 3(B)), the IC50 value of PPDS was 19.01 μM, which was lower than IC50 values of free SAHA (23.76 μM), herein, the PPD was proved to enhance the cytotoxicity of free SAHA as a biocompatible vector of HDACi.

### 4.6. Apoptosis and cell cycle arrest

**Figure 4.**
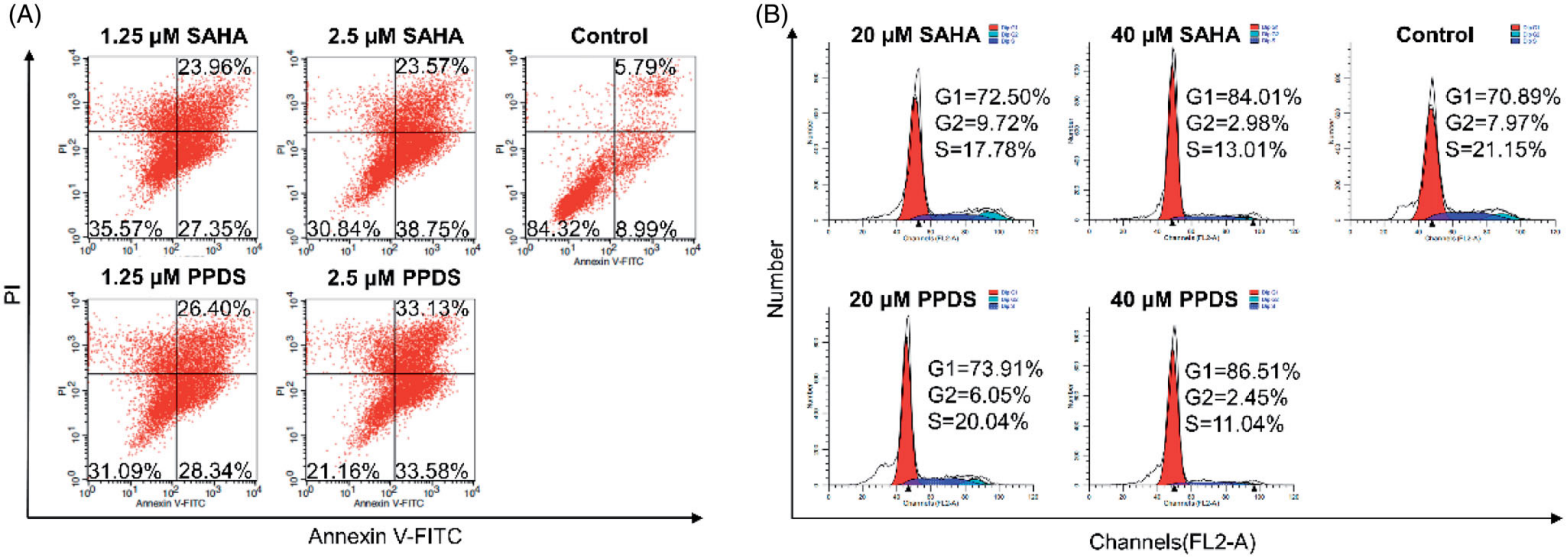
The mechanism of tumor cells death induced by PPDS. The apoptosis of HepG2 cells (A) and cell cycle arrest of B16-F10 cells (B) detected by flow cytometry after treated with PPDS and free SAHA.

The antitumor mechanism of PPDS was investigated by apoptosis and cell cycle arrest. Cell apoptosis was detected by flow cytometry (AnnexinV-FITC/PI Analysis Kit) (Figure 4(A)). Compared to blank group control, both PPDS and free SAHA (1.2 and 2.5 μM) caused HepG2 cells apoptosis in a dose-dependent manner. Specifically, PPDS (1.25 μM) showed an apoptotic cell proportion of 54.74%, which was higher than that of free SAHA (51.31%); in contrast, PPDS (2.5 μM) showed an apoptotic cell proportion of 66.71%, which was higher than that of free SAHA (62.32%), indicating polymerized SAHA (PPDS) as a HDACi prodrug could enhance the therapeutic effect due to its intensive and lysosome-responsive release in cytoplasm.

Tumor cell cycle arrest was inspected using flow cytometry (EZ Cell TM Cell Cycle Analysis Kit) (Figure 4(B)). We have established PPDS group, SAHA group (20 and 40 μM), and blank control group, respectively. Compared with the blank control group (G1 = 70.89%), the B16-F10-cells treated with both free SAHA and PPDS (20 and 40 μM) were inclined to be blocked inG1 phase, which was in accordance with literature report (Wang et al., 2012; Bernhart et al., 2017). Moreover, more tumor cells treated with PPDS were blocked in G1 phase than that treated with free SAHA (G1_PPDS, 20 μM_ =73.91%, G1_PPDS, 40 μM_ =86.51%, G1_free SAHA, 20 μM_ =72.50%, G1_free SAHA, 40 μM_ =84.01%), and the blocking effect was dose-dependent.

All the above results were basically consistent with the results of cytotoxicity data measured by CCK-8 assay, indicating that compared with free SAHA, PPDS could be uptaken into tumor cells with a higher efficiency to exert its antitumor effect with inducing cell apoptosis and blocking the cell cycle in G1 phase.

### 4.7. *In vivo* antitumor efficacy

**Figure 5.**
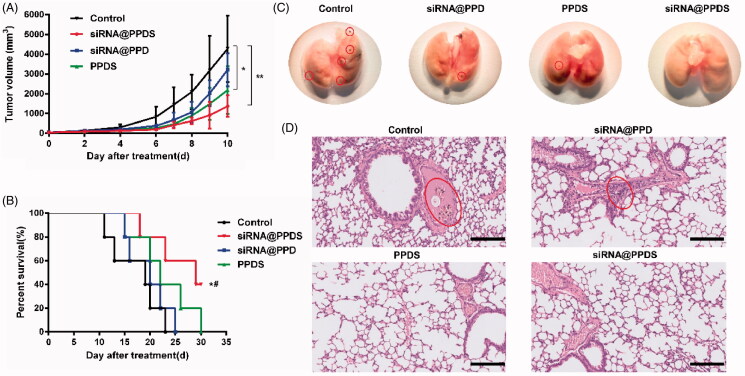
Antitumor efficacy of siRNA@PPDS on mice bearing B16-F10 melanoma *in vivo*. (A) tumor volume curve (*p < .05, **p < .01, compared with control group); (B) survival curves of C57BL/6 mouse model (control vs siRNA@PPDS, *p = 0.0357; siRNA@PPD versus siRNA@PPDS, ^#^*p* = .0471); (C) the photographs of lung tissues with metastatic nodules of the first dead mice bearing B16-F10 melanoma in each group after treatment; (D) Microscopic images obtained by H&E staining of the lung sections from the first dead mice bearing B16-F10 melanoma in each group after treatment (×40, scale bar of 100 μm).

To evaluate the therapeutic effect of combined PD-L1-KD and HDACIs *in vivo*, an antitumor study was performed on B16-F10tumor bearing C57BL/6 mouse model. The mice were randomly divided into four groups and then treated with saline, PPDS, siRNA@PPD and siRNA@PPDS, respectively. The mice were administrated drugs in five cycles at a time interval of 2 days. Since the first dose administrated, the tumor volumes were measured every day up to 10 days. As shown in Figure 5(A), compared with saline group, siRNA@PPDS significantly inhibited tumor growth (*p* < .01), while siRNA@PPD or PPDS showed relatively slight inhibition of tumor growth (*p* > .05 or *p* < .05), hinting that the combination of PD-L1-KD and HDACIs in micelles dramatically inhibited tumor growth, much more efficient than PD-L1-KD alone. Furthermore, the statistical analysis of the mouse survival curve (Figure 5(B)) showed that compared with the control group or the siRNA@PPD group, the survival time of mice in the siRNA@PPDS group (*p* < .05) was statistically different, also indicating that siRNA@PPDS showed remarkable antitumor activity.

Metastasis is known as the leading threat to tumor patients' survival, so the antimetastasis effect of siRNA@PPDS was probed into by observing metastatic nodules in mice bearing melanoma. As shown in Figure 5(C), no lung metastatic nodules were found in melanoma-bearing mice treated with siRNA@PPDS, whereas the saline group had the most lung metastatic nodules, followed by siRNA@PPD or PPDS. Moreover, the lung metastasis inhibition effect of siRNA@PPDS was further confirmed by H&E staining of the lungs (Figure 5(D)), in which lung metastatic nodules were observed in the sectioned field of lungs treated with saline and siRNA@PPD. All findings above indicated that the combination of PD-L1-KD and HDACIs in micelles can be a potential treatment for inhibiting tumor growth and may play a role in inhibiting tumor metastasis.

## 5. Conclusion

In summary, we successfully fabricated a lysosome activable multifunctional micelle for effective co-delivery of SAHA and siRNA-PD-L1, which aims at combining PD-L1-KD induced cancer immunotherapy and HDACIs chemotherapy to exert a synergetic therapeutic effect on tumor proliferation and metastasis. siRNA@PPDS demonstrated excellent cellular uptake for B16-F10 cells; also, SAHA and siRNA-PD-L1 could be spontaneously released into the cytoplasm under the acidic and lipase conditions in the lysosome, thereby inducing tumor cell apoptosis, blocking the cell cycle in G1 phase and silencing PD-L1 proteins. Furthermore, tumor volumes, survival period, and lung section from melanoma-bearing mice *in vivo* indicated the siRNA@PPDS could significantly inhibit tumor growth and pulmonary metastasis comparing with siRNA-PD-L1 or SAHA alone. All in all, with a controlled release of siRNA-PD-L1 and SAHA spontaneously into the cytoplasm of tumor cells, siRNA@PPDS demonstrated an outstanding therapeutic effect on mice bearing melanoma *in vitro* and *in vivo*, revealing that by comprehensively activating the immune system, lysosome activable polymeric vorinostat encapsulating PD-L1KD for the combination therapy of PD-L1-KD and HDACIs can be an effective strategy to reverse the unresponsiveness of immune checkpoint inhibitors and a promising treatment to inhibit tumor growth and metastasis in clinic.

## Supplementary Material

Supplemental Material
