# Supplementary material for: Lysosome activable polymeric vorinostat encapsulating PD-L1KD for a combination of HDACi and immunotherapy
Source: Drug Deliv. 2021 May 26;28(1):963–72. doi: 10.1080/10717544.2021.1927246 (PMC8158275; doi:10.1080/10717544.2021.1927246)
Supplement: Supplemental Material [file IDRD_A_1927246_SM3390.docx]

**Supplementary material**s

**1. Synthesis of PPDS and determination of SAHA drug loading**

**1.1 Synthesis of L-aspartate benzyl ester-N-carboxylic acid anhydride (BLA-NCA)**

L-Aspartic acid-4-benzyl ester (BLA, 4.7 g, 0.02 mol) was dissolved in 60 mL of anhydrous THF, then triphosgene (6.0 g, 0.02 mol) was added and the mixture was magnetically stirred at 60℃. After the solution became clear, it was concentrated by rotary steamer. When the composite became viscous, it was recrystallized with chloroform and n-hexane at 0°C to provide BLA-NCA as white crystal solid.

**1.2** **Synthesis of polyethylene glycol - block- poly (aspartic acid benzyl ester) (PEG-b-PBLA)**

PEG-NH_2_ (0.4 g) and BLA-NCA (3.57 g) were dissolved in 10 mL or 30 mL of anhydrous DCM respectively, then the solution was mixed and reacted for 72h at room temperature under N_2_ protection. The crude was concentrated by rotary steamer and re-crystallized with DCM and ether (Et_2_O) at 0°C to provide PEG-b-PBLA as a yellow powder.

**1.3** **Synthesis of polyethylene glycol-b-polyasparagine (PEG-b-P[Asp(DET)_n_], abbreviated as** **PPD)**

PEG-*b*-PBLA (0.4g) was dissolved in 10 mL of DMF, 8 mL of anhydrous DET was stirred and reacted at 35℃for 24h, then the mixture was dialyzed against deionized water (pH=5) for 1 day and lyophilized for 3 days to obtain the product PPD (^1^HNMR in Figure S1-2A).

**1.4 Synthesis of PPDS**

100 mg SAHA was dissolved in DMF and 62 mg CDI in DMF was immediately added. The mixture was stirred magnetically for 1h under the protection of N_2_ at room temperature, then 186 mg PEG113-P[Asp(DET)_n_] was added to the above reaction solution, and the mixture was magnetically stirred for 24h under the protection of N_2_ at 35℃. The crude was dialyzed against deionized water of pH 5 for 1 day and lyophilized for 3 days to provide the final product PPDS. The conjugation of PPD with SAHA was verified by ^1^HNMR spectrum (^1^HNMR in Figure S1-2B).

**1.5** **Determination of drug loading of SAHA**

SAHA loading rate was determined by the ratio of the peak areas of the corresponding groups on the ^1^HNMR spectrum. The corresponding relationship between the number of H and the peak area was as follows: PEG (4 H; peak area = 1), DET (8 H; peak area = 1.13), benzene ring on SAHA (5 H; peak area = 0.48). According to the formula: 4* n_PEG_/8*n_DET_=1/1.13, 4* n_PEG_/5*n_SAHA_=1/0.48, n_PEG_= 113 (n is the degree of polymerization), n_DET_ and n_SAHA_ were calculated as 64 and 43, respectively, therefore the binding rate of SAHA/PPDS was as follows: M_SAHA*_43/M_PPDS_=264*43/30310=0.37g/g.

**2. Establishment of standard curve of SAHA**

4mg SAHA was dissolved in 40 mL solvent (0.4 mL DMSO + 39.6 mL pH 7.4 的PBS). The solution was diluted to obtain SAHA solutions with concentrations of 100, 90, 80, 70, 60, 50, 40, 30, 20, 10, 5, 2.5 μg/mL. The SAHA solution was scanned by an ultraviolet spectrophotometer at 200-800 nm and the maximum absorption wavelength of SAHA was measured to be 240 nm. Then the absorbance of the above-mentioned SAHA solution was measured at a wavelength of 240 nm, finally, the standard curve was established based on the relationship between concentration and absorbance (Figure S2).

**Fig S1-1** Synthesis process of PPDS

**Figure S1-2** The ^1^HNMR of PPD **(A)** and PPDS **(B).**

**Figure S2** Standard curve of SAHA.

**Figure S3 (A)** Cytotoxicity of siRNA@PPD or siRNA-NC@PPD to B16 cells. **(B)** Cytotoxicity of PPD to HepG2 cells. **(C)** Cytotoxicity of PPD to B16 cells.
